# Molecular epidemiology of *Brucella abortus* in Shandong, China: high-resolution insights from combined MLVA-16 and core genome SNP analysis

**DOI:** 10.3389/fmicb.2025.1695815

**Published:** 2025-10-23

**Authors:** Chanjuan Huangfu, Xiujun Ma, Junjie Fan, Kuo Han, Ti Liu, Zengqiang Kou, Yan Li

**Affiliations:** ^1^School of Public Health and Health Management, Shandong First Medical University, Jinan, China; ^2^Shandong Centre for Disease Control and Prevention, Jinan, China; ^3^Weifang Centre for Disease Control and Prevention, Weifang, China

**Keywords:** *Brucella abortus*, molecular epidemiology, genotyping, MLVA, cgSNP

## Abstract

**Introduction:**

The recent identification of *Brucella abortus* in human clinical samples from Shandong, China, highlights an ongoing zoonotic threat.

**Methods:**

We characterized 12 *B. abortus* strains isolated from human patients since 2021 using a combination of conventional biotyping, Multiple Locus Variable-number Tandem Repeat Analysis (MLVA), and core-genome SNP (cgSNP) analysis.

**Results:**

Epidemiological data indicated that infections primarily occurred in middle-aged men with occupational livestock exposure. Molecular typing revealed biovar 3 as the predominant type (91.7%), dominated by MLVA-8 genotype 36 and its corresponding MLVA-11 genotype 72 (66.7%). MLVA-16 distinguished 12 unique genotypes. The phylogeny based on cgSNP classified the strains into clades C1 (11 bv. 3 strains) and C2 (one bv. 1 strain). Within clade C1, nine strains in subclade C1-III exhibited ≤119 SNP differences, eight of which formed a local clonal transmission chain (≤52 SNPs) and shared MLVA-11 genotype 72. Subclade C1-I contained two strains with novel genotypes resulting from variations at the Bruce18 and Bruce43 loci. The sole C2 strain differed by only 3 SNPs from the A19 vaccine strain, suggesting a potential vaccine-related origin. Genetic links were also identified with strains from other Chinese provinces, among them Heilongjiang and Inner Mongolia, as well as from several countries, including Mongolia and Russia.

**Discussion:**

These findings revealed a complex epidemiological pattern in Shandong, primarily characterized by local transmission chains with occasional external introductions, provided a scientific basis for targeted brucellosis control strategies.

## 1 Introduction

Brucellosis, caused by the genus *Brucella*, is a globally significant bacterial zoonosis and is listed as a priority for control by the Food and Agriculture Organization (FAO) and the World Health Organization (WHO) (Cutler et al., 2005). In China, human brucellosis remains a major public health problem, causing substantial economic losses and threatening both physical and mental health. The prevention and control of brucellosis is a national strategic priority. The Ministry of Agriculture and Rural Affairs included it in its plan for infectious diseases requiring control and eradication in 2012. Furthermore, the Chinese government launched a five-year control action plan in 2022, underscoring the urgency of its prevention and control efforts. Shandong Province, as a major livestock production region in China, serves as a crucial frontline defence in the national control of brucellosis. The province has reported a persistently high number of human cases in recent years, with an annual average exceeding 2,700 (Yu et al., 2023). Between 2021 and 2024, cumulative reported cases reached 13,096, with the annual incidence rate consistently maintaining a high level between 3.13 per 100,000 and 3.32 per 100,000. This presents a severe and persistent challenge to local public health authorities.

*Brucella* spp. are pathogenic bacteria with cross-host adaptability, capable of spreading among natural hosts through direct or indirect contact and infecting other susceptible animals (Pappas et al., 2006; De Massis et al., 2019). Among the 12 known *Brucella* species, the *B.melitensis*, *B.abortus*, and *B.suis* pose the greatest threat to human health, but their pathogenicity varies. *B.melitensis* and *B.suis* strains are the most virulent, while *B.abortus* has relatively milder pathogenicity (Alton and Jones, 1967). Cattle brucellosis is primarily caused by *B. abortus*, leading to abortion, infertility, and reduced milk production in cattle, and causing significant economic losses to farmers by reducing livestock productivity and reproductive capacity (Tian et al., 2024). In humans, infection manifests as irregular fever, arthritis, and other clinical symptoms (Dean et al., 2012). Through biotyping schemes, *B. abortus* has been classified into seven distinct biovars. Based on multi-locus sequence typing (MLST-21) research, *B. abortus* can be divided into three major evolutionary clades globally. The early-branching clade A/B strains are entirely of African origin, while the widely distributed clade C is further divided into two major clades, C1 and C2, which represent the main drivers of recent global spread of *B. abortus* (Whatmore et al., 2016). Among these, C1 is highly associated with bv. 3 and is primarily distributed across the Eurasian continent; C2 is dominated by bv. 1 and exhibits global widespread distribution. This MLST-based clade framework has become a crucial foundation for studying the population genetics and geographical distribution of this bacterial species.

Although the macro-epidemiological profile of human brucellosis in Shandong Province has been outlined, the molecular characteristics of the causative *Brucella* pathogens remain largely unexplored (Yu et al., 2023). Specifically, in contrast to the relatively well-studied *B. melitensis*, data on the population structure, genetic diversity, and transmission dynamics of *B. abortus* isolates from human clinical cases in this region are strikingly scarce (Li et al., 2024). This lack of high-resolution molecular data impedes a thorough understanding of the origins of local outbreaks and hinders the development of targeted control strategies.

Effective control of brucellosis relies on precise monitoring and high-resolution methods to identify sources of infection and transmission pathways. Although MLST provides an important foundation for population studies, its limited resolution still limits its application in molecular epidemiology. MLVA provides higher resolution and standardized typing schemes by detecting copy number variations in tandem repeat loci, making it suitable for tracking regional transmission chains. However, its resolution remains limited in precisely tracing the origin and transmission of outbreak strains. Advances in molecular technology have driven the development of typing methods toward genome-based technologies. In recent years, molecular typing methods based on WGS technology (such as cgSNP and wgSNP) have become the mainstream direction due to their ability to provide the highest resolution of genotypic information and their applicability to detailed genetic evolution and transmission studies (Holzer et al., 2021; Edao et al., 2023).

Therefore, this study investigated the molecular epidemiology of *B. abortus* from human cases in Shandong Province between 2021 and 2024. We used MLVA-16 and cgSNP analysis to investigate the genetic characteristics, population structure, and geographical distribution of clinical isolates. This work provides the first comprehensive molecular dataset for *B. abortus* in the region, offering scientific evidence to improve surveillance and enable targeted interventions for brucellosis control in Shandong and throughout China.

## 2 Materials and methods

### 2.1 Strain sources and patient inclusion criteria

All *Brucella* isolates in this study were obtained from the human brucellosis surveillance system in Shandong Province, China, between 2021 and 2024. Strain inclusion was based on the following criteria: isolates were derived from patients meeting the confirmed case definition of brucellosis according to the Chinese Health Industry Standard (WS 269–2019), which requires a clear epidemiological history, typical clinical manifestations, a positive Rose Bengal plate test (RBT), and a standard tube agglutination test (SAT) titer of ≥1:100++ (China’s National Health Commission, 2019).

Blood samples from eligible patients were cultured using biphasic blood culture bottles. Blood culture bottles that signaled positive but from which no *Brucella* colonies could be isolated on Columbia blood agar plates, or that were identified as non-*Brucella* species, were excluded from the study. Samples that yielded no visible bacterial growth on the solid agar phase were excluded. Culture bottles with visible growth were transported under standardized protocols by local CDC Centres to the Shandong Provincial CDC for further identification and bacterial isolation. All isolates were confirmed as *B. abortus* by the provincial reference laboratory through biochemical profiling, PCR assays, and molecular typing. Following this protocol, a total of 12 clinical *B. abortus* isolates were successfully collected and included in this study.

### 2.2 *Brucella* isolation and DNA extraction

After enrichment of 5–10 mL blood samples in blood culture bottles, a 100 μL aliquot of the enriched culture was inoculated onto Columbia blood agar plates and incubated at 37 °C under 5% CO₂ for 48–72 h. Typical *Brucella* colonies, characterized by being moist, round, slightly raised, smooth, and opalescent, were observed on Columbia blood agar plates (Supplementary Figure S1). *Brucella* colonies were subcultured to obtain pure isolates. A single colony was suspended in 200 μL of sterile physiological saline and inactivated at 80 °C for 90 min. The genomic DNA, extracted with the TIANamp Bacterial DNA Kit (TianGen, Beijing, China), was used for subsequent molecular identification and whole-genome sequencing.

### 2.3 Identification and typing of strains

Phenotypic characterization and biotyping were performed using standard *Brucella* identification methods according to the Chinese Health Industry Standard (China’s National Health Commission, 2019). The CO₂ requirement was assessed by comparing bacterial growth on Columbia blood agar after 48 h of incubation at 37 °C under both aerobic and 5% CO₂ conditions. Slide agglutination tests were carried out using monospecific antisera against A, M, and R antigens to evaluate antigenic reactivity. Dye inhibition susceptibility was determined using discs impregnated with thionin (20 μg/mL) and basic fuchsin (20 μg/mL); inhibition zones were recorded following incubation at 37 °C under 5% CO₂ for 48 h.

Molecular identification of all 12 isolates was performed on the extracted DNA using the BCSP31-PCR assay for genus confirmation (Fekete et al., 1990), and AMOS-PCR for species and biovar discrimination (Bricker and Halling, 1994). The primer sequences, detailed PCR reaction mixtures, and thermal cycling conditions used in this study are provided in Supplementary Table S1. The amplified products were analyzed by 1.5% agarose gel electrophoresis and imaging (Supplementary Figure S2).

### 2.4 Whole genome sequencing

Following molecular identification, the genomic DNA of 12 isolates was subjected to paired-end sequencing (PE150) on the Illumina NovaSeq X Plus platform. The raw data output for each sample exceeded 1 Gb, with an average sequencing depth of 100×. Raw reads were first evaluated comprehensively using FastQC v0.11.9 for quality assessment. Subsequently, Fastp v0.21.0 was employed for strict quality control and filtering to generate high-quality clean data along with relevant quality metrics. *De novo* genome assembly was performed using SPAdes v3.15.5 based on the cleaned reads. The assembly quality was evaluated using key assembly metrics, including N50, N90, GC content, and number of scaffolds. The genome assembly metrics for all 12 isolates are summarized in Supplementary Table S2.

### 2.5 Core genome SNP genotyping

A core-genome phylogenetic analysis was conducted using the Snippy v4.6.0. Following alignment of all isolate sequences to the *B. abortus* 2308 reference genome (GenBank accession numbers NC_007618 and NC_007624), a high-quality core-genome alignment was generated.

### 2.6 Phylogenetic and diversity analysis

Molecular characterization was performed based on whole-genome sequencing data. The assembled genomes of the 12 isolates were imported into CLC Genomics Workbench 23.0.3 (QIAGEN, Germany) for MLVA-16 analysis. Using standard *Brucella* MLVA primer sequences as probes, target loci were identified and extracted from the whole genomes. The copy numbers of tandem repeats at the 16 VNTR loci were precisely determined by comparing the amplified fragment lengths with the known sizes of repeat units. The resulting MLVA-16 profiles were analyzed using BioNumerics 8.0 (Applied Maths, Belgium). An Unweighted Pair Group Method with Arithmetic Mean (UPGMA) dendrogram was constructed by clustering the study isolates with 112 *B. abortus* strains originating from China. In addition, a Minimum Spanning Tree (MST) was generated incorporating the study isolates and 286 *B. abortus* strains isolated globally since 2015.

For higher-resolution evolutionary analysis, a cgSNP phylogenetic tree was constructed. The 12 Shandong isolates were analyzed together with 390 globally representative *B. abortus* reference genomes obtained from the NCBI database. The core-genome alignment and SNP extraction were performed using the workflow described in Section 2.5, yielding a core genome SNP alignment file. A maximum-likelihood (ML) phylogenetic tree was constructed from this alignment using MEGA X (Mega Limited, New Zealand), with nodal support assessed by 1,000 bootstrap replicates. To enhance the clarity and phylogenetic resolution of the tree, strains within the same clade that exhibited identical core-genome sequences were filtered out. This process resulted in a refined, high-quality dataset comprising the 12 local Shandong isolates and 141 globally representative strains, which was used to infer the final SNP-based phylogeny.

### 2.7 Geographic information mapping

The geographic distribution of the studied strains was visualized using ArcGIS 10.8 (ESRI, USA). The map was projected in the WGS 1984 geographic coordinate system. Geographic coordinates (longitude and latitude) for each isolate, representing the residential locations of the patients they originated from, were obtained from epidemiological records. The resulting point layer map clearly displays the locations of the 12 isolates, with points symbolized according to key attributes such as biovar, MLVA genotype, and year of isolation.

## 3 Results

### 3.1 Epidemiological and clinical characteristics of patients infected with *Brucella abortus*

**Figure 1:**
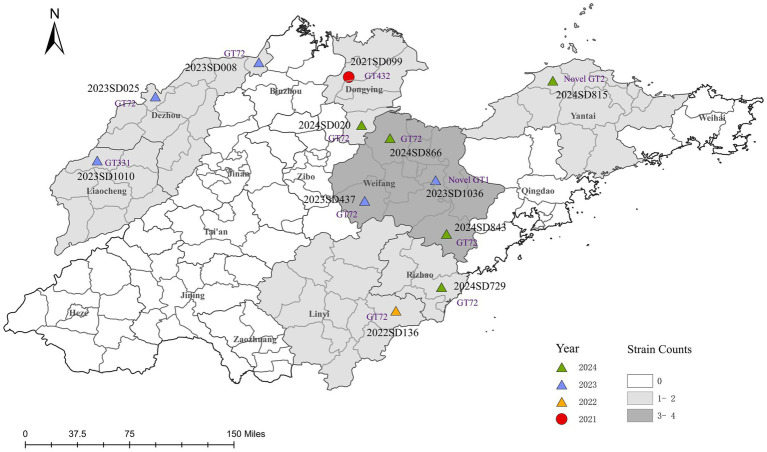
Spatiotemporal distribution of clinical *B. abortus* isolates from human cases in Shandong Province, China, 2021–2024. The map shows the geographical distribution of 12 *B. abortus* clinical isolates. Points are colored according to the year of isolation and shaped according to the biovar. Each point is labeled with the corresponding strain ID and its MLVA-11 genotype (novel genotypes are denoted as Novel GT1 and Novel GT2). The background colour of prefecture-level administrative regions is graded according to the total number of isolates collected. The base map was created using ArcGIS 10.8 software.

Epidemiological characteristics were analyzed for 12 laboratory-confirmed human cases of *B. abortus* infection identified in Shandong Province between 2021 and 2024 (Table 1). Most patients were male (9/12, 75.0%) with a median age of 48 years (range: 25–58); the majority (10/12, 83.3%) were between 40 and 59 years old. The annual number of cases increased over time, with one case each in 2021 and 2022, and five cases in both 2023 and 2024. Geographically, cases were distributed across seven cities (Figure 1), with the highest number reported in Weifang (4/12, 33.3%), followed by Dezhou and Dongying (2 cases each, 16.7%), and single cases in Linyi, Liaocheng, Rizhao, and Yantai. Occupational exposure was the primary transmission route (11/12, 91.7%), predominantly through cattle or sheep rearing (9 cases). Only one case was attributed to foodborne transmission. The most characteristic clinical symptoms were arthralgia (91.7%), fever (75.0%), and hyperhidrosis (66.7%), consistent with the classic presentation of brucellosis.

**Table 1:** Epidemiological and clinical characteristics of patients infected with *B. abortus* in Shandong Province, China.

Strain ID	Gender	Age	City	Occupation	Primary exposure route	Clinical symptoms
2021SD099	Male	40	Dongying	Farmer	Sheep rearing	Fever, Hyperhidrosis, Arthralgia
2022SD136	Male	50	Linyi	Farmer	Cattle rearing	Fever, Hyperhidrosis, Arthralgia
2023SD008	Male	41	Dezhou	Farmer	Cattle rearing	Fever, Hyperhidrosis, Arthralgia
2023SD025	Female	58	Dezhou	Farmer	Cattle rearing	Fever, Hyperhidrosis, Arthralgia
2023SD437	Male	41	Weifang	Farmer	Cattle rearing	Arthralgia
2023SD1010	Female	38	Liaocheng	Farmer	Livestock trading	Fever, Arthralgia
2023SD1036	Male	46	Weifang	Farmer	Cattle rearing	Arthralgia
2024SD020	Male	25	Dongying	Veterinarian	Veterinary practices	Fever, Arthralgia
2024SD729	Male	51	Rizhao	Farmer	Cattle rearing	Fever, Hyperhidrosis
2024SD815	Female	56	Yantai	Farmer	Consumption of contaminated mutton	Hyperhidrosis, Arthralgia
2024SD843	Male	53	Weifang	Farmer	Sheep rearing	Fever, Hyperhidrosis, Arthralgia
2024SD866	Male	54	Weifang	Farmer	Sheep rearing	Fever, Hyperhidrosis, Arthralgia

### 3.2 Strain identification and biovar distribution

Through traditional phenotypic identification combined with BCSP31-PCR and AMOS-PCR molecular identification, it was confirmed that all 12 isolates analyzed in this study were *B. abortus*. (Supplementary Figure S2). Biotyping revealed a clear temporal and geographical pattern (Figure 1). Only one isolate, recovered from Dongying in 2021, was identified as *B. abortus* bv. 1. The remaining eleven isolates (91.7%), collected from the other six cities between 2022 and 2024, were all identified as bv. 3, confirming it as the predominant biovar. To date, no *B. abortus* strains have been reported from the other nine prefecture-level cities in Shandong Province.

### 3.3 Results of MLVA-16 genotyping

MLVA genotyping analysis was performed on 12 strains of *B. abortus* in Shandong. The results showed that among the MLVA-8 genotypes, 11 strains (91.7%) were known genotypes, with genotype 36 (4-5-3-12-2-2-3-1) being the predominant genotype, accounting for 8 strains (66.7%). Other genotypes included 112 (*n* = 1), 116 (*n* = 1), and 235 (*n* = 1). One strain (2024SD729, Yantai) was identified as a novel genotype due to variations in the number of repeats at the Bruce43 locus. In MLVA-11 typing, 10 strains (83.3%) were known genotypes, with genotype 72 (corresponding to genotype 36 in MLVA-8) also accounting for 66.7% (8/12). Other genotypes included genotype 331 (*n* = 1) and genotype 432 (*n* = 1). Two strains (2023SD1036, Weifang; 2024SD815, Yantai) were identified as novel strains due to variations in the number of repeats at the Bruce18 locus. MLVA-16 further improved resolution, with 12 strains identified as 12 unique genotypes, demonstrating high genetic diversity among the strains.

**Figure 2:**
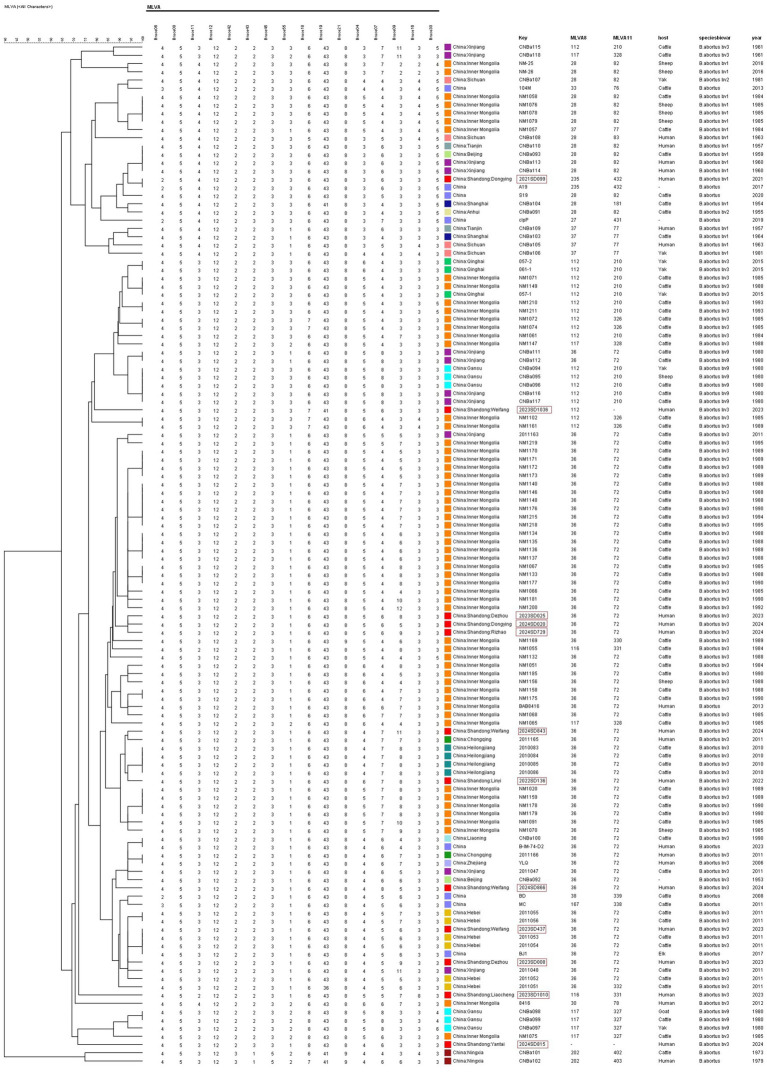
UPGMA cluster analysis of *B. abortus* isolates from China based on MLVA-16 genotyping. The dendrogram was constructed using UPGMA, analyzing the genetic relationships among 124 strains. This set included 12 isolates from this study and 112 reference strains from various regions across China. The tree illustrates the distribution of the Shandong isolates within the national genetic context.

UPGMA clustering analysis was performed on 112 representative strains of *B. abortus* from other regions of China downloaded from MLVAbank (Figure 2; Supplementary Table S3). One strain (2023SD437, Weifang) shared the same MLVA-16 genotype with three domestic strains (two from Hebei). One strain (2021SD099, Dongying) shared the same MLVA-16 genotype with one A19 strain isolated domestically. The remaining 10 Shandong strains had independent MLVA-16 genotypes and did not share any with other domestic strains. In the domestic database, genotype 36 was the predominant type, with genotypes 112, 116, and 235 also present, among which 235 was the least common.

**Figure 3:**
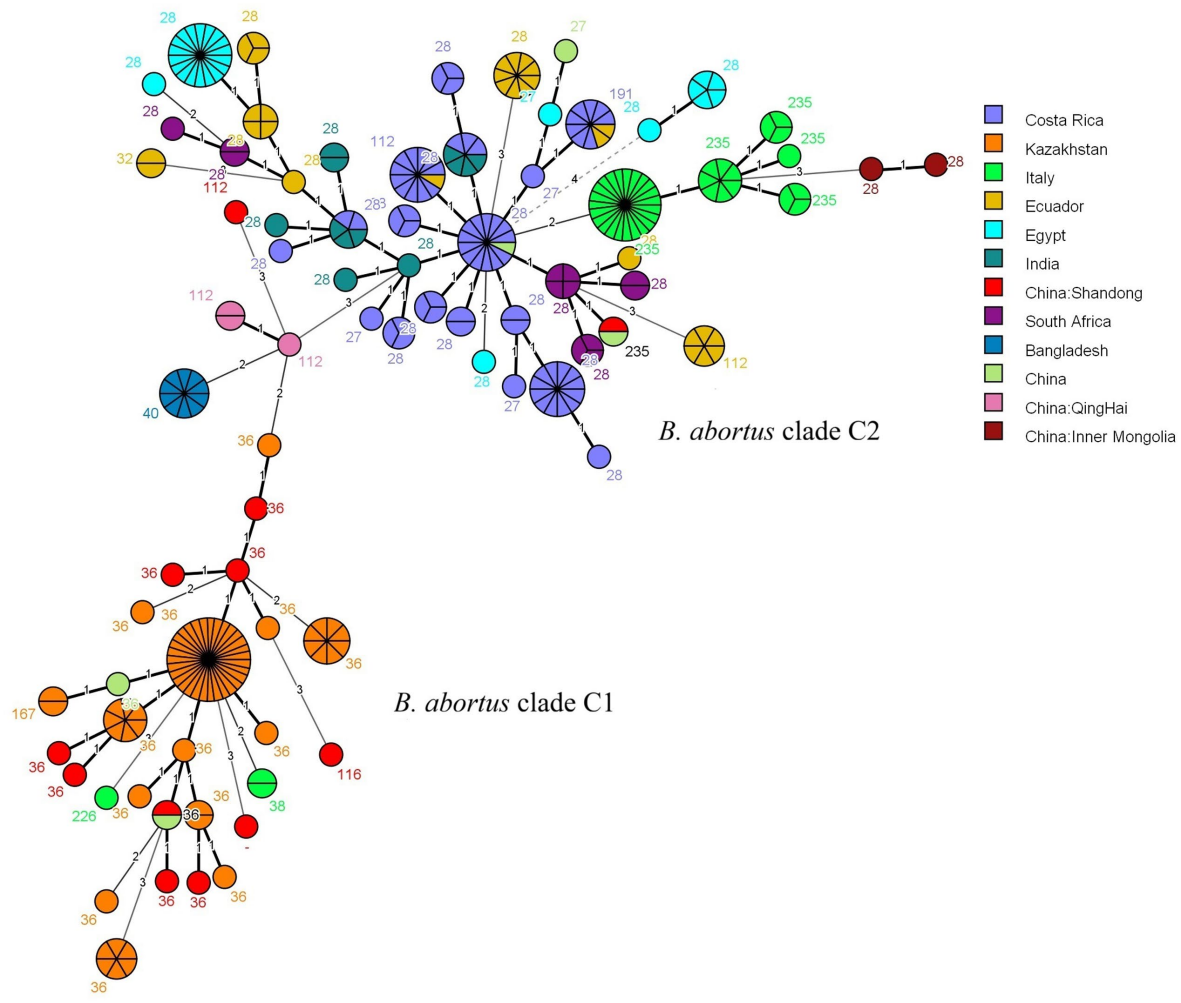
MST of *B. abortus* isolates from worldwide based on MLVA-16 genotyping. The analysis included 286 strains: 12 isolates from this study (highlighted in red) and 274 global reference strains collected since 2015. The reference strains are coloured by their country of origin. Based on the MLST typing information available in the MLVAbank database for the reference strains, the two main clusters are labeled as the *B. abortus* clade C1 and *B. abortus* clade C2 evolutionary clades to indicate the phylogenetic position of the Shandong isolates. Each node represents a unique MLVA-16 genotype, with its size proportional to the number of isolates. The number adjacent to each node corresponds to its MLVA-8 genotype code.

The MLVA-16 data from this study were analyzed using MST with global strains from the MLVAbank database since 2015 (Figure 3; Supplementary Table S3). Ten strains from Shandong (MLVA-8: 8 strains of genotype 36, 1 strain of genotype 116, and 1 strain of a novel genotype) were distributed in the clade C1 and clustered with strains from Kazakhstan and other countries. The remaining 2 strains (MLVA-8: 1 strain of genotype 112 and 1 strain of genotype 235) were distributed in the clade C2, with the genotype 112 strain clustering most closely with strains from Qinghai, China, and the genotype 235 strain exhibiting the closest genetic affinity to strains from South Africa.

### 3.4 cgSNP analysis results

**Figure 4:**
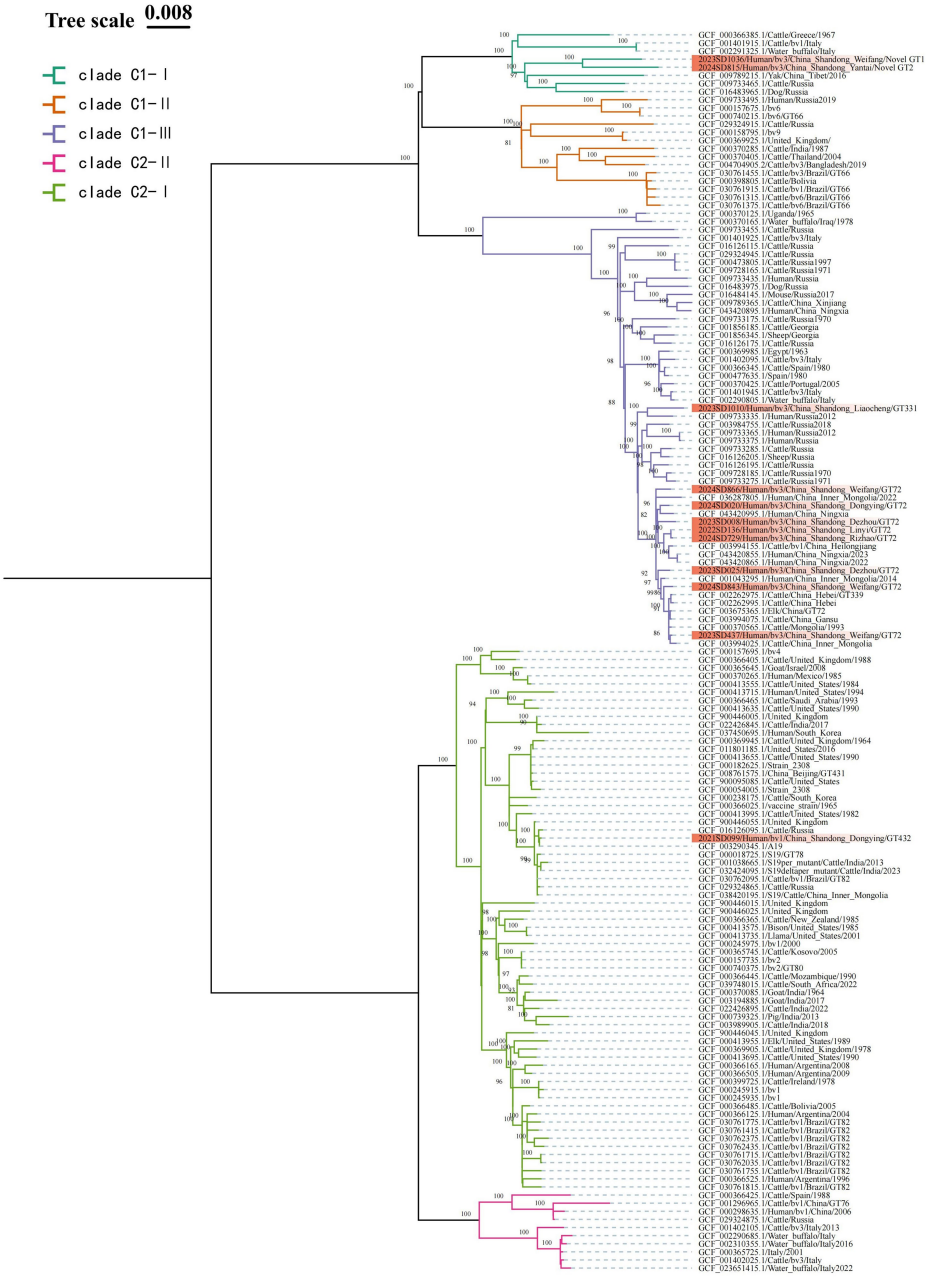
ML phylogenetic tree of *B. abortus* strains based on cgSNP. To enhance phylogenetic resolution, strains within the same clade possessing identical core-genome sequences are filtered out. The final dataset used for phylogenetic reconstruction consist of 12 local Shandong isolates (highlighted in red) and 141 globally representative strains. Nodal support is assessed with 1,000 bootstrap replicates.

The MST analysis based on MLVA-16 initially divided the strains into two main clusters. According to the MLST typing information of reference strains in the MLVAbank database, these two clusters were correspondingly labeled as the C1 and C2 evolutionary clades of *B. abortus*; the subsequent cgSNP phylogenetic analysis adhered to this clade framework and performed higher-resolution analysis within it. To validate and further resolve this population structure, cgSNP analysis was performed based on WGS data from the 12 Shandong isolates and global *B. abortus* reference strains from NCBI, and a phylogenetic tree was constructed (Figure 4). The results showed that all strains were clearly divided into two major clades, C1 and C2. Clade C1 included 11 Shandong isolates (all bv. 3), which were further divided into three subclades: C1-I, C1-II, and C1-III. Subclade C1-I contained two Shandong strains (2023SD1036, Weifang; 2024SD815, Yantai), which differed by 186 SNPs. These two strains were classified as novel genotypes by MLVA-11 typing and clustered with a Tibetan yak-derived strain isolated in Tibet, China, in 2016 (SNP differences: 323 and 345, respectively). Their closest foreign relative was a cattle-derived strain from Greece (SNP differences: 310 and 332, respectively), forming a cluster with Italian and Russian strains. Subclade C1-III included 75% of Shandong strains (9/12), with 1 strain from 2022, 4 strains from 2023, and 4 strains from 2024. The maximum pairwise SNP distance among these nine strains was less than 119, which, in the context of the global phylogeny, indicates a close genetic relationship. Among them, the Liaocheng strain 2023SD1010 was genetically closest to the Russian human-derived strain from 2012, with only 87 SNP differences. The remaining 8 strains were genetically closest to human- or cattle-derived strains from Heilongjiang, Ningxia, Hebei, Inner Mongolia, and Gansu provinces in China, with SNP differences within 66 SNPs, and also showed close phylogenetic relationships with cattle-derived strains from Mongolia. Notably, the Linyi strain (2022SD136) and Rizhao strain (2024SD729) differed by only 3 SNPs, suggesting they may represent the same clone or share a common infection source. Subclade C1-II was not detected among the Shandong isolates in this study.

Clade C2 contained only one Shandong isolate (2021SD099, bv. 1, Dongying), which further branches into two subclades: C2-I, and C2-II. This Shandong strain resides within C2-I, differing from the A19 vaccine strain by only 3 SNPs, indicating extremely close genetic proximity. Its SNP differences with other vaccine strains (such as S19 and 2308) are also within 30 sites. In the phylogenetic tree, strains clustered around this isolate were predominantly bv. 1 strains. Subclade C2-II was not detected among the Shandong isolates in this study.

## 4 Discussion

This study provided the first systematic analysis of the epidemiological patterns of *B. abortus* derived from human cases in Shandong Province. By integrating epidemiological and molecular genotyping data, we revealed that its transmission dynamics are characterized by a core of local transmission chains, along with extensive genetic linkages to strains from other Chinese provinces and internationally. These findings provide key molecular epidemiological evidence for enhanced disease surveillance and precise source tracing in the region. In 2021, *B. abortus* was first isolated from human cases in Shandong, and a total of 12 isolates have been obtained to date. This number is significantly lower than that of *B. melitensis* isolates, consistent with previous studies indicating that *B. abortus* accounts for a very low proportion of human brucellosis outbreaks, and that *B. melitensis* remains the dominant species in human cases in Asian countries such as Iran (Vergnaud et al., 2018; Khan et al., 2021; Dadar et al., 2023). This phenomenon may be attributed to the relatively lower potential virulence of *B. abortus* compared to *B. melitensis* (Kneipp et al., 2019).

All 12 patients with *B. abortus* infection in this study had clear epidemiological exposure histories, indicating a primarily local transmission pattern. Patients were predominantly male (75.0%) and aged 40–59 years (83.3%), consistent with the demographics of livestock workers (Liang et al., 2020). In terms of exposure routes, except for one foodborne case, all other cases were related to occupational contact with livestock, including nine cases of family farming, one veterinarian, and one livestock trader. Our findings indicated multiple pathways of transmission in Shandong Province: family farming serves as the main transmission route, infection in a veterinarian reflects occupational risk, livestock trading may expand the spread, and the foodborne case indicates a potential threat in the local food chain. These findings are consistent with the known transmission routes of brucellosis through direct contact or consumption of contaminated animal products (Gwida et al., 2010; Pappas, 2010). The main clinical symptoms were arthralgia (91.7%), fever (75.0%), and hyperhidrosis (66.7%), matching the typical manifestations of brucellosis. Spatiotemporal analysis showed a recent clustering trend, with 83.3% (10/12) of isolates obtained between 2023 and 2024, and 33.3% (4/12) of cases concentrated in Weifang. This clustering suggested an active local transmission chain in Weifang and surrounding areas. The developed livestock industry and high-density cattle and sheep farming in this region are likely contributing factors. High-density farming increases contact among animals, raising the likelihood of *Brucella* spread within herds, and thereby increasing the risk of human exposure to the source of infection (Yu et al., 2023).

Strain identification revealed that 91.7% (11/12) of the isolates were *B. abortus* bv. 3, consistent with the global predominance of this biovar (Ma et al., 2020). To unravel the genetic characteristics, we employed MLVA, a key technique in *Brucella* molecular epidemiology that effectively identifies subtle genetic variations among strains and is suitable for analyzing transmission chains at regional and even global levels (Wareth et al., 2020; Holzer et al., 2022). In recent years, the in silico MLVA typing based on whole-genome sequences has also been widely adopted. MLVA analysis revealed the genetic characteristics of the Shandong strains at multiple loci (Dadar and Alamian, 2025). On one hand, a widely disseminated dominant clone was identified, with genotype 36 (MLVA-8) and its corresponding genotype 72 (MLVA-11) being predominant (8/12, 66.7%). This genotype has been persistently prevalent in China since the 1980s and has also been reported in Eurasian countries such as Kazakhstan and Portugal, suggesting close genetic links between the Shandong epidemic strains and Eurasian strains (Ferreira et al., 2012; Jiang et al., 2013; Shevtsov et al., 2015). On the other hand, the population exhibited high diversity. Higher-resolution MLVA-16 distinguished the 12 strains into 12 unique genotypes. Furthermore, novel genotypes were detected arising from variations in the number of repeats at the Bruce43 and Bruce18 loci. These sites were previously considered highly stable in Chinese *B. melitensis*; their mutation suggests ongoing region-specific microevolution within the local endemic population (Kattar et al., 2008; Jiang et al., 2011; Mirkalantari et al., 2021).

Clustering analyses based on domestic and international strains provided multidimensional insights into the origins and genetic background of *B. abortus* in Shandong Province (Sayour et al., 2020). UPGMA clustering using a domestic strain database showed that Shandong strains possessed both unique and shared characteristics with strains from other regions. Most isolates (10/12, 83.3%) exhibited distinct MLVA-16 genotypes, indicating a significant local genetic background. However, a few strains shared genotypes with isolates from other provinces; for example, the Weifang strain 2023SD437 shared an identical genotype with two strains from Hebei, suggesting inter-regional transmission. The Dongying strain 2021SD099 matched a domestic A19 vaccine strain, providing a direction for further traceback investigation. MST analysis based on global MLVA data further clarified the population structure of Shandong strains on a broader evolutionary scale. All strains clustered within clade C, which could be divided into two subclades, C1 and C2. Subclade C1 was the dominant group, comprising ten bv. 3 strains. These strains formed a tight cluster with isolates from Kazakhstan and other countries, indicating close genetic relationships. This genetic connection was likely driven by the combined effects of geographical proximity and historical ruminant trade along routes akin to the grassland silk road, factors jointly known to facilitate the regional spread of *Brucella* (Liu et al., 2020; Shevtsov et al., 2023). In contrast, subclade C2 contained only two Shandong strains but demonstrated a complex genetic background, with members originating from various regions in China, such as Inner Mongolia and Qinghai, as well as other countries including South Africa and Italy. This pattern suggests a history of transregional or even international transmission for this subclade. The presence of the rare bv. 1 strain in Shandong is of particular importance for surveillance and early warning.

Phylogenetic analysis based on cgSNP provided higher-resolution validation and refinement of the genetic structure revealed by MST. Both methods classified the Shandong strains into two primary lineages, clade C1 and clade C2, with no detection of clade A or B. This population structure is consistent with findings from studies in Kazakhstan and other Asian countries. However, minor discrepancies in the classification of individual strains were observed between the two methods (MST: C1 = 10, C2 = 2; cgSNP: C1 = 11, C2 = 1), underscoring the superior resolution of cgSNP and highlighting the necessity and complementarity of high-resolution methods in molecular epidemiological investigations. cgSNP analysis confirmed clade C1 as the dominant lineage. The nine *B. abortus* bv. 3 strains within this clade exhibited a maximum pairwise SNP distance of less than 119, indicating their close genetic relatedness within the context of the global phylogeny and suggesting descent from a common ancestor. Notably, eight strains from Liaocheng and other areas showed ≤52 SNP differences and shared the MLVA-11 genotype 72, strongly supporting their belonging to an active clonal transmission chain (Jackson et al., 2016). This clone showed close phylogenetic relationships with strains from Heilongjiang and Inner Mongolia in China, as well as from Mongolia and Russia, implying a possible common regional origin. This pattern of regional transmission is consistent with findings from Sichuan Province, where *Brucella* strains frequently share genetic links with those from neighboring provinces and countries (Li et al., 2025). The sole *B. abortus* bv. 1 strain was unambiguously assigned to clade C2 by cgSNP analysis. This strain differed from the standard A19 vaccine strain by only 3 SNPs. When combined with its MLVA-16 profile and the patient’s occupation as a livestock farmer, these findings suggested that the strain is probably related to the A19 vaccine, likely resulting from exposure during vaccination procedures. This finding underscored that although the A19 vaccine has been widely and safely used in China since the 1940s, strict adherence to biosafety protocols by personnel is essential to prevent accidental infection (Wang et al., 2020; Zhou et al., 2022).

Our results clearly demonstrated the complementary value of MLVA and cgSNP typing techniques. MLVA is effective for the initial identification of dominant genotypes and potential transmission clusters on a regional scale (Zhao et al., 2021), such as the successful identification of the genotype 36 clonal group widely distributed across the province. In contrast, cgSNP provides higher resolution for analyzing finer genetic variation, offering superior capability in confirming genetic relatedness within clones, enabling precise traceback, and correcting biases inherent in conventional typing methods (Holzer et al., 2021; Kydyshov et al., 2022; Zhu et al., 2024). For instance, although the Linyi strain 2023SD136 and the Rizhao strain 2024SD729 had different MLVA-16 genotypes, they showed only 3 SNP differences, suggesting they likely originated from the same transmission event and providing conclusive evidence of inter-city spread within the province. Furthermore, one strain initially classified into C2 by MST analysis was reassigned to clade C1 based on cgSNP, more accurately reflecting its true genetic ancestry. The two novel MLVA-11 genotypes were independently distributed within subclade C1-I in the cgSNP analysis, confirming them as genuine genetic variants potentially representing either new introductions or local evolutionary events. Therefore, combining the broad screening capacity of MLVA with the high-resolution traceback capability of cgSNP provided a more comprehensive molecular epidemiological basis for elucidating pathogen transmission dynamics (Li et al., 2024).

## 5 Conclusion

This study presented the first comprehensive molecular epidemiological investigation of *B. abortus* in Shandong Province, China, by integrating high-resolution MLVA-16 and cgSNP analysis. Our results confirmed the dominant prevalence of *B. abortus* bv. 3 and revealed the sporadic occurrence of a bv. 1 strain genetically related to the A19 vaccine strain, indicating the coexistence of natural transmission and potential vaccine-related events. cgSNP analysis provided high-resolution genetic insights, revealing closely related clusters among the predominant bv. 3 strains. This finding indicated the presence of localized transmission chains within the broader context of sporadic distribution, which is crucial for understanding the micro-evolution and spread of *B. abortus* in endemic regions.

The successful application of the combined MLVA-cgSNP approach offered a powerful strategy for bacterial genotyping and traceback investigations. From a public health perspective, the genetic linkages identified between Shandong strains and those from other provinces and countries underscore the transboundary nature of brucellosis transmission. This highlighted the necessity for enhanced regional collaboration and coordinated cross-jurisdictional control measures. Furthermore, the detection of a vaccine-like strain emphasized the importance of continuous monitoring and prudent vaccine management in the field.

In summary, this study filled a critical knowledge gap regarding the molecular epidemiology of *B. abortus* in Shandong Province. The findings provided a scientific foundation for evidence-based prevention and control strategies, contributing to the optimization of brucellosis surveillance networks and the development of more effective public health interventions in China, ultimately aiming to reduce the disease burden in both animal and human populations.

## Data Availability

The data presented in the study are deposited in the NCBI repository, accession number PRJNA1310245.

## Supplementary material

SUPPLEMENTARY FIGURE S1 Typical colony morphology of *Brucella abortus* on Columbia blood agar after 48-72 hours of incubation.

SUPPLEMENTARY FIGURE S2 Gel electrophoresis results of BCSP31-PCR and AMOS-PCR for the identification and biotyping of *Brucella* isolates.

SUPPLEMENTARY TABLE S1 Oligonucleotide primers, PCR reaction mixtures, and thermal cycling conditions used in this study. This table is provided as a separate Excel file (Supplementary Table S1.xlsx) containing the following three sheets: Sheet 1 (Primers): Oligonucleotide primers used in this study. Sheet 2 (Reaction_Mixture): Detailed PCR reaction components and their final concentrations. Sheet 3 (Cycling_Conditions): Thermal cycling parameters for both PCR assays.

SUPPLEMENTARY TABLE S2 Quality metrics and assembly statistics for the whole-genome sequencing data. The detailed content of Table S2 is provided as a separate Excel file (Supplementary Table S2.xlsx).

SUPPLEMENTARY TABLE S3 Complete MLVA-16 profiles for the strains analyzed in this study. Sheet 1 (UPGMA): Contains the Shandong isolates and Chinese reference strains used for UPGMA analysis (related to Figure 2 in the main text). Sheet 2 (MST): Contains the Shandong isolates and global reference strains used for MST analysis (related to Figure 3 in the main text). The detailed content is provided as a separate Excel file (Supplementary Table S3.xlsx).

SUPPLEMENTARY TABLE S4 Molecular and genomic characteristics of *Brucella abortus* strains from human cases in Shandong Province, China (2021-2024). The detailed content of Table S4 is provided as a separate Excel file (Supplementary Table S4.xlsx). The table includes strain identifiers, genomic accession numbers, geographical origins, collection years, biovar classification, and complete MLVA genotyping profiles.
