# Supplementary figures and images for: Molecular epidemiology of Brucella abortus in Shandong, China: high-resolution insights from combined MLVA-16 and core genome SNP analysis
Source: Front Microbiol. 2025 Oct 23;16:1695815. doi: 10.3389/fmicb.2025.1695815 (PMC12588999; doi:10.3389/fmicb.2025.1695815)

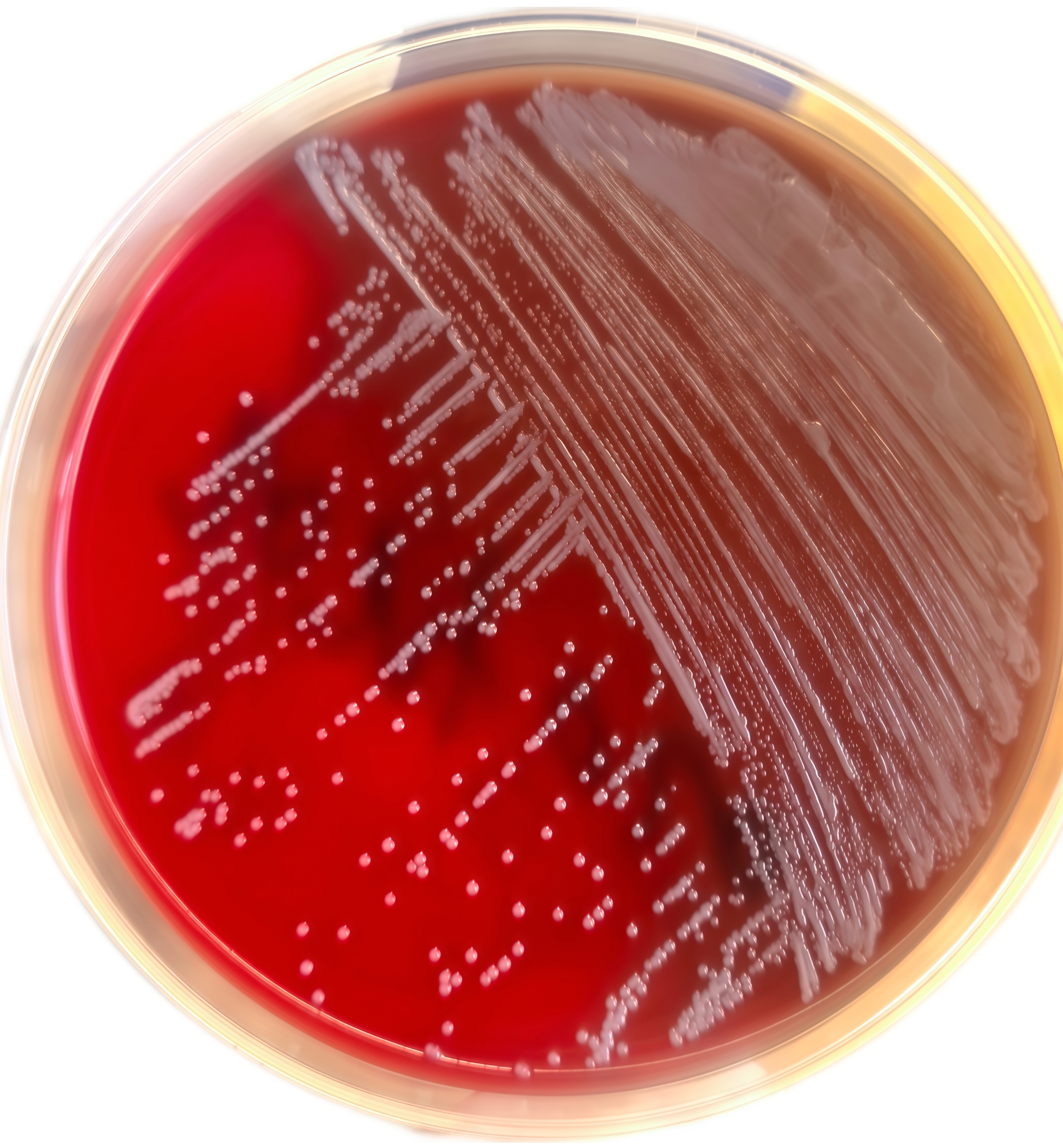

Supplement: SUPPLEMENTARY FIGURE S1 — Typical colony morphology of Brucella abortus on Columbia blood agar after 48-72 hours of incubation. [file Image_1.jpeg]

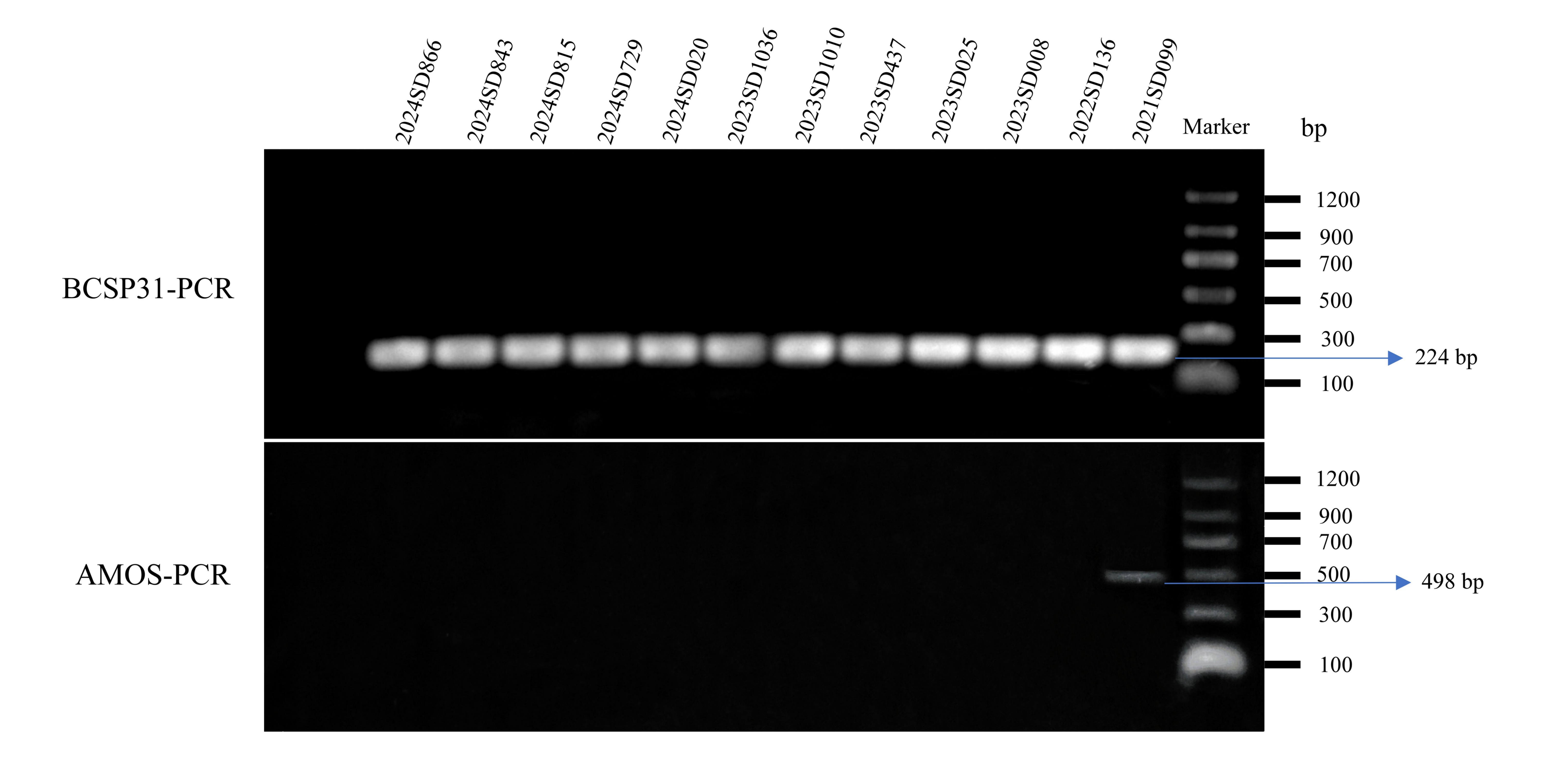

Supplement: SUPPLEMENTARY FIGURE S2 — Gel electrophoresis results of BCSP31-PCR and AMOS-PCR for the identification and biotyping of Brucella isolates. [file Image_2.jpeg]
